# Distinct requirements for Pho, Sfmbt, and Ino80 for cell survival in *Drosophila*

**DOI:** 10.1093/genetics/iyab096

**Published:** 2021-07-19

**Authors:** Pavel Elizarev, Katja Finkl, Jürg Müller

**Affiliations:** Laboratory of Chromatin Biology, Max-Planck Institute of Biochemistry, Martinsried 82152, Germany

**Keywords:** Polycomb, Pho, Sfmbt, Ino80, apoptosis, *Drosophila*

## Abstract

The *Drosophila* proteins Pleiohomeotic (Pho) and its paralog Pho-like (Phol) are the homologs of the mammalian transcription factor YY1. Pho and Phol are subunits of the Polycomb group protein complex PhoRC and they are also stably associated with the INO80 nucleosome remodeling complex. *Drosophila* lacking both Pho and Phol arrest development as larvae with small misshaped imaginal discs. The basis of this phenotype is poorly understood. We find that in *pho phol* mutant animals cells retain the capacity to proliferate but show a high incidence of apoptotic cell death that results in tissue hypoplasia. Clonal analyses establish that cells stringently require Pho and Phol to survive. In contrast, the PhoRC subunit Sfmbt and the ATP-dependent nucleosome remodeling factor Ino80 are not essential for cell viability. Pho and Phol, therefore, execute their critical role for cell survival through mechanisms that do not involve Sfmbt function or INO80 nucleosome remodeling.

## Introduction 

Genetic studies in *Drosophila* originally identified *Polycomb* (*Pc*) and several other genes because of homeotic phenotypes that are caused by widespread misexpression of multiple HOX genes (Lewis 1978; Duncan 1982; Jurgens 1985; reviewed in Kassis *et al.* 2017). To date, mutations in 17 different *Drosophila* genes are known to cause this phenotype, and these genes are therefore classified as Polycomb group (PcG) genes (reviewed in Kassis *et al.* 2017). Biochemical studies revealed that the proteins encoded by PcG genes are the subunits of four distinct multiprotein complexes: PolycombRepressive Complex 1 (PRC1), PRC2, Polycomb Repressive Deubiquitinase (PR-DUB), and Pho-Repressive Complex (PhoRC) (Shao *et al.* 1999; Czermin *et al.* 2002; Müller *et al.* 2002; Klymenko *et al.* 2006; Scheuermann *et al.* 2010). PRC1, PRC2, and PR-DUB modify the chromatin of PcG target genes through enzymatic but also through nonenzymatic activities to bring about transcriptional repression by mechanisms that are only partially understood (reviewed in Kassis *et al.* 2017; Bracken *et al.* 2019; Yu *et al.* 2019). PhoRC, in contrast, is not known to modify nucleosomes but its subunit Pleiohomeotic (Pho), the *Drosophila* homolog of the mammalian transcription factors YY1, is the only PcG protein with sequence-specific DNA-binding activity (Brown *et al.* 1998; Klymenko *et al.* 2006).

Genetic, genomic, biochemical, and structural studies have provided compelling evidence that Pho, together with its binding partner protein Sfmbt, play an essential role for recruitment of PRC1 and PRC2 to PcG target genes (Wang *et al.* 2004; Mohd-Sarip *et al.* 2005; Klymenko *et al.* 2006; Oktaba *et al.* 2008; Schuettengruber *et al.* 2009; Strübbe *et al.* 2011; Alfieri *et al.* 2013; Kahn *et al.* 2014; Frey *et al.* 2016). However, unlike any of the other PcG proteins, Pho and its redundantly acting paralog Pho-like (Phol) are also required for survival of somatic cells (Brown *et al.* 2003; Klymenko *et al.* 2006). In particular, if clones of *pho phol* double mutant cells are induced in somatic tissues of heterozygous animals, the mutant cell clones are lost from the tissue after a few cell generations (Klymenko *et al.* 2006). Neither Sfmbt nor any of the other PcG mutants show comparably compromised cell viability. It, therefore, appears that Pho and Phol also function in processes that do not require the other components of the PcG system.

A possible link of Pho to other processes is suggested by the observation that Pho is not only present in PhoRC but also co-purifies with the INO80 nucleosome remodeling complex in *Drosophila* embryos (Klymenko *et al.* 2006). This association is conserved in mammalian cells, where YY1 also exists in a stable assembly with the INO80 complex (Cai *et al.* 2007; Wu *et al.* 2007). Ino80, the catalytic subunit of the INO80 nucleosome remodeling complex (Eustermann *et al.* 2018), participates in a plethora of different chromatin-modifying processes, including the spacing of nucleosome or the exchange of the histone variant His2Av in *Drosophila* or of its orthologue H2AZ in mammals with canonical histone H2A (Papamichos-Chronakis *et al.* 2011; Udugama *et al.* 2011; Krietenstein *et al.* 2016; Brahma *et al.* 2017). Could the function of Pho and Phol in an Ino80-regulated process explain the impaired viability of *pho phol* double mutant cells? Previous studies have reached conflicting conclusions about the requirement of the Ino80 protein in *Drosophila*. Bhatia *et al.* (2010) reported a purported *Ino80* null mutation where homozygotes for this mutation invariably die as late-stage embryos. In contrast, Bashirullah and colleagues reported that homozygotes for another purposed *Ino80* null mutation develop into morphologically normal and viable but sterile adults (Neuman *et al.* 2014). The reason for this discrepancy has remained unclear.

Here, we investigated how tissue development and cell proliferation are compromised in *Drosophila* mutants lacking Pho and Phol, Sfmbt, or Ino80. Our analyses uncover that cells lacking Pho and Phol protein retain the capacity to proliferate normally but show a strongly increased incidence of apoptotic cell death. We show that *Sfmbt* or *Ino80* protein null mutants, or *Sfmbt Ino80* double mutants do not show this cell death phenotype. This highlights that Pho and Phol ensuring cell survival through a mechanism that does not require the chromatin-modifying activities of the PcG machinery or nucleosome remodeling by INO80.

## Materials and methods

### *Drosophila* strains

The following *Drosophila* strains were used for this study:

*w^1118^* (used as *wildtype* reference)

*pho^1^/unc13^act-GFP^*

*phol^81A^ FRT2A/TM3, 2xTb^1^-RFP*

*w; phol^81A^ FRT2A/TM6B; pho^1^/unc13^act-GFP^*

*y w hs-Flp; hs-nGFP FRT2A; pho^1^/unc13^act-GFP^*

*y w; Sfmbt^1^ FRT40A/CyO, ubi-GFP*

*w; Ino80^KO^/TM6B, ubi-GFP*

*Sfmbt^1^ FRT40A; Ino80^KO^/T(2;3)TSTL14, SM5: TM6B*

*pho^1^* is a null allele caused by insertion of a 4.5 kb Doc retrotransposon within codon 272 (GenBank sequence AE014135.4 coordinates 1,174,346–1,174,348 for chromosome 4) upstream of the zinc finger coding region of the *pho* gene (Brown *et al.* 1998).

*phol^81A^* is a null allele obtained by imprecise excision of a P-element. In the *phol^81A^*, part of the P-element was deleted along with the entire *phol*-coding region (Brown *et al.* 2003). The deletion encompasses nucleotides 9,452,378–9,456,100 on chromosome 3 L (AE014296.5 coordinates).

*Sfmbt^1^* is a null allele obtained by homologous recombination and insertion of the *mini-white* gene cassette into *Sfmbt*, thereby disrupting the *Sfmbt* open reading frame and deleting 13,173,712–13,173,766 on chromosome 2 L (coordinates according to AE014134.6) (Klymenko *et al.* 2006). In addition, *Sfmbt^1^* carries an inversion of the *Sfmbt* 3’ coding sequences downstream of the *mini-white* insertion cassette and its coding potential was thereby destroyed.

*Ino80^KO^* is a null allele generated via CRISPR/Cas9 genome editing in this study. For molecular details, see below.

### Generation of an *Ino80^KO^* null mutation

To generate the *Ino80^KO^* null allele by CRISPR/Cas9 genome editing, a 971 base pair (bp) long 5’ homology arm and 978 bp long 3’ homology arm were cloned into the pHD-DsRed-attP vector (Gratz *et al.* 2014). Guide RNA target sequences close to the 5’ homology arm (TGGCGTTGGATGCCGATATG) and to the 3’ homology arm (TTACTGTCTACGAGAGCCGG) were cloned into the pCFD3 vectors (Port *et al.* 2014). Plasmids were co-injected into embryos from a *nos-Cas9* strain (Port *et al.* 2014, Bloomington *Drosophila* Stock Center stock 54591). Two independent chromosomes carrying *Ino80^KO^* were isolated and found to have the same molecular lesion, with a deletion of nucleotides 19,403,153–19,398,737 on chromosome 3 R (GenBank sequence AE014297.3 coordinates), the deleted sequence starts with ACTGTGCGAATGGCGTTGGA… and ends with …TTAACAGCCGCCGATACGGT (Supplementary Figure S1).

### Western blot analysis of larval extracts

For analysis of Pho, Sfmbt, or Ino80 protein levels in wild-type and mutant larvae, appropriate diploid tissues were hand-dissected from third instar larvae, homogenized in SDS sample buffer, briefly sonicated, centrifuged, and the supernatant was analyzed on an SDS polyacrylamide gel and processed for western blotting. The following antibodies were used: rabbit anti-Pho_324__–__520_ (1:10000) (Klymenko *et al.* 2006), rabbit anti-Sfmbt_531__–__980_ (1:1000) (Klymenko *et al.* 2006), rabbit anti-Ino80_1261__–__1510_ (1:1000) (Klymenko *et al.* 2006), rabbit anti-Ogt_1__–__300_ (H-300) (1:5000) (Santa Cruz Biotechnology sc-32921), rabbit anti-Caf1-55_full-length_ (1:50000) (Gambetta *et al.* 2009).

### Clonal analysis and immunostaining procedures

Mutant clones in imaginal discs were generated and analyzed by immunostaining and confocal microscopy as described (Beuchle *et al.* 2001). For analysis of *phol^81A^* mutant clones in *pho^1^* homozygotes, *y w flp122; hs-nGFP FRT2A; pho^1^/unc13^act-GFP^* virgin females were crossed to *w; phol^81A^ FRT2A/TM6B; pho^1^/unc13^act-GFP^* males. In all cases, larvae of the appropriate genotypes were identified using the appropriate GFP and or *Tb* markers. The following antibodies were used for immunostainings: mouse anti-Abd-B clone 1A2E9 (1:200) (Developmental Studies Hybridoma Bank), mouse anti-Antp clone 8C11 (1:100) (Developmental Studies Hybridoma Bank), rabbit anti-cDcp-1 (1:300) (#9578; Cell Signalling), rabbit anti-H3S10ph (1:500) (#06-570; Merck-Millipore). DNA was visualized using staining by Hoechst 33342 with 1 µg/ml concentration.

### Whole mount preparations of adults

Freshly hatched adults were stored in 70% ethanol and for 16 hours incubated in PBST (0,1% Triton) before mounting in Hoyer’s medium.

### Image analysis

Quantification of cDcp-1- and H3S10ph-positive cells was performed by measuring the density of fluorescently labeled nuclei using ImageJ (Schneider *et al.* 2012), complemented with all the default plugins provided by FIJI (Schindelin *et al.* 2012) and the additional updates provided by the ImageScience site.

In the case of H3S10ph-positive nuclei, the area of the wing imaginal discs was measured and nuclei were counted in ImageJ software (Find Maxima command, prominence 100) after being enhanced (FeatureJ Laplacian command, smoothing parameter equal to 2 µm). A script was written to repeat the same analysis on all images. In the case of cDcp-1-positive nuclei, the same procedure was applied but the nuclei were counted manually using the Multi-point tool.

### Data availability

*Drosophila* strains generated in this study are available upon request. Supplemental material available at G3. 

## Results

**Figure 1:**
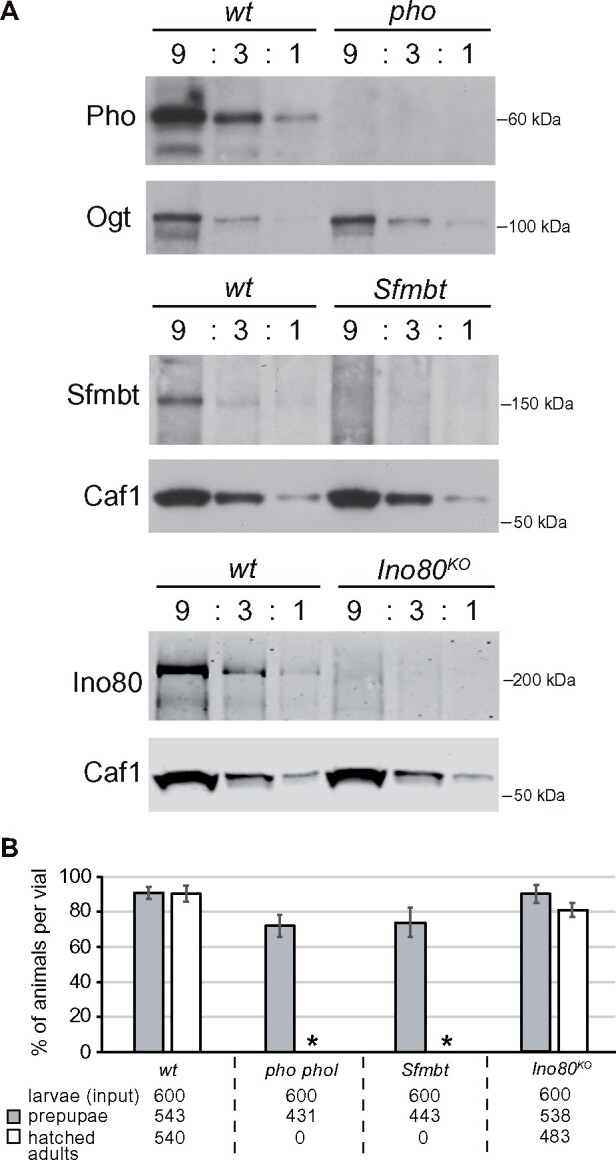
Lack of zygotic expression of Pho and Phol, or of Sfmbt results in developmental arrest during puparium formation. (A) Maternally deposited Pho, Sfmbt and Ino80 proteins are undetectable in late-stage *pho*, *Sfmbt* or *Ino80^KO^* mutant larvae, respectively. Western blots on serial dilutions (9:3:1) of total extracts from tissues of third-stage larvae that were wildtype (*wt*), or homozygous for *pho^1^* (*pho*, top), for *Sfmbt^1^* (*Sfmbt*, middle), or for *Ino80^KO^* (bottom) and derived from heterozygous parents in all cases. In the case of *Sfmbt^1^* and *pho^1^* mutant larvae, imaginal disc and CNS tissues were used for extract preparation; in the case of *Ino80^KO^* mutant larvae, extracts were prepared from wing, haltere and third leg imaginal discs tissues. Top: the western blot membrane was probed with antibodies against Pho and, as control, the membrane was simultaneously probed with antibodies against Ogt. Middle: Western blot membrane was probed with antibodies against Sfmbt and Caf1-55 (Caf-1). Bottom: Western blot membrane was probed with antibodies against Ino80 and Caf1-55. (B) Viability of *Drosophila* larvae that were *wild-type* (*wt*), *pho phol* double mutant, *Sfmbt* mutant or *Ino80^KO^* mutant; in all cases, the homozygous mutants were derived from parents that were heterozygous for the indicated mutations. For each genotype, 600 late second/early third-instar larvae (input) were collected and reared in batches of 100 larvae in six separate vials. In each vial, the percentage of animals that formed prepupae (gray bar) and eclosed from the pupal case (white bar) was determined. Histogram bars represent the mean and standard deviation of these percentages in individual vials. Note, *pho phol* double mutant and *Sfmbt* mutant larvae all invariably arrested development as early prepupae without undergoing metamorphosis and no adults eclosed (asterisks). Note that *Ino80^KO^* homozygotes survive into viable adults at a frequency comparable to wildtype.

To investigate the phenotype of animals lacking Pho or Phol, or both Pho and Phol, or Sfmbt, we used animals that were homozygous for previously described null mutations *pho^1^*, *phol^81A^*, or *Sfmbt^1^*, respectively (Brown *et al.* 1998, 2003; Klymenko *et al.* 2006; see Materials and Methods)*.* Because Pho, Phol, Sfmbt are all essential for development of the germline and formation of a fertilized zygote (Brown *et al.* 2003; Klymenko *et al.* 2006), it was in each case only possible to analyze mutants derived from heterozygous parents. We shall refer to the analyzed homozygous mutants as *pho* mutants, *phol* mutants, *pho phol* double mutants, and *Sfmbt* mutants. As previously reported, in *pho phol* double mutants or *Sfmbt* mutant animals, maternally deposited Pho and Phol, or Sfmbt protein, respectively, likely permit these animals to complete embryogenesis and develop into the larval stages (Brown *et al.* 2003; Klymenko *et al.* 2006). Because of turnover and dilution due to cell division, these maternally deposited protein products are then however no longer present in diploid tissues from third instar larvae. As illustrated in Figure 1A, Pho protein was undetectable in extracts from imaginal disc and CNS tissues dissected from *pho* single mutant larvae (cf. Brown *et al.* 2018) and, similarly, Sfmbt was undetectable in these tissues in *Sfmbt* mutant larvae (Figure 1A).

To study the phenotype of *Ino80* null mutants, we used CRISPR/Cas9 genome editing to generate a molecularly defined *Ino80^KO^* allele by deleting the chromosomal region encoding amino acid residues 1–1245 of the 1638 codon open reading frame of Ino80 (Supplementary Figure S1A). The *Ino80^KO^* deletion, therefore, lacks the region encoding the entire Ino80 N-terminus and the two lobes of the Ino80 ATP-dependent helicase domain but the deletion does not disrupt the other genes encoded in the intron regions of the *Ino80* locus (Supplementary Figure S1A). *Ino80^KO^* homozygotes developed into viable adults that were morphologically indistinguishable from wild-type flies (Supplementary Figure S1B, Figure 1B). Whereas *Ino80^KO^* homozygous males were fertile, the *Ino80^KO^* homozygous females were completely sterile, suggesting that Ino80 is essential for development of the female germline. Together, these observations on the phenotype of *Ino80^KO^* homozygotes corroborate the *Ino80* mutant phenotype that Bashirullah and colleagues had reported using the *Ino80^psf25^* allele (Neuman *et al.* 2014). It should be noted that Bashirullah and colleagues found that only 20% of *Ino80^psf25^* homozygotes develop into adults (Neuman *et al.* 2014), whereas, in our analyses, the fraction of *Ino80^KO^* homozygotes developing into adults was only very slightly lower than in wildtype (Figure 1B). Considering that the chromosome carrying *Ino80^psf25^* had been isolated following chemical mutagenesis by EMS (Neuman *et al.* 2014), whereas the *Ino80^KO^* mutation had been genetically engineered in an isogenized homozygous viable chromosome, it seems likely that differences in the genetic background account for this difference in survival into adults. Finally, we note that the requirement of Ino80 for development of the female germline again only permitted the analysis of *Ino80^KO^* homozygotes derived from heterozygous parents. As expected, Ino80 protein was undetectable in imaginal disc tissues dissected from *Ino80^KO^* homozygous mutant third instar larvae (Figure 1A).

In a next step, we analyzed the stage of lethality and investigated possible developmental delays during the growth of *pho phol*, *Sfmbt* and *Ino80^KO^* mutant larvae. As previously reported (Brown *et al.* 2003; Klymenko *et al.* 2006), *pho phol* and *Sfmbt* mutants arrested development during the early phase of puparium formation and we found no animals that would develop past this stage (Figure 1B). Quantification of larval viability showed that the majority of *pho phol* or *Sfmbt* mutant animals complete larval development and do form a puparium (Figure 1B). However, the *pho phol* mutant animals were considerably delayed in their development and reached the late third larval instar stage only about 192 hours after egg lay (AEL). Even though the fraction of *Ino80^KO^* homozygotes that developed into adults was similar to wild-type, we found that the *Ino80^KO^* homozygotes eclose with a delay of about one day compared to wild-type flies (Figure 1B, Supplementary Figure S1C).

**Figure 2:**
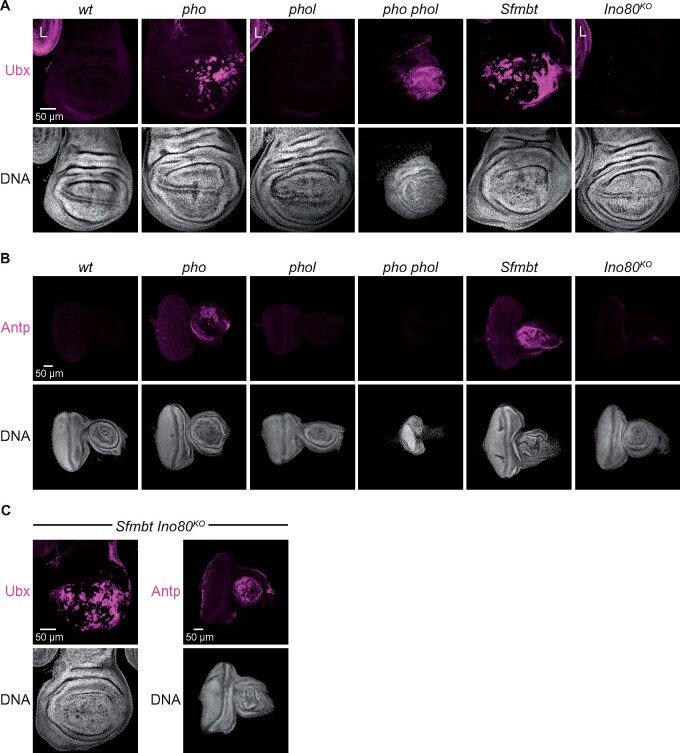
Larvae lacking zygotic expression of PhoRC show misexpression of multiple HOX genes. (A) Wing imaginal discs of third-instar larvae from animals with the indicated genotype, stained with antibody against Ubx protein and Hoechst (DNA) to label all nuclei. Note the reduced size of the wing disc in the *pho phol* double mutant. Ubx protein is misexpressed in the pouch portion of the wing discs in *pho*, *pho phol*, and *Sfmbt* mutants. In the wing discs of *phol* or *Ino80^KO^* single mutants, no misexpression of Ubx could be detected; the wild-type pattern of Ubx expression in metathoracic leg discs (L) visible in these two genotypes and in the wildtype (*wt*) serves as reference. (B) Eye antennal imaginal discs of the same genotypes like in (A), stained with antibody against Antp protein and Hoechst (DNA). Note, Antp protein is misexpressed in the antennal lob of the discs from *pho* and *Sfmbt* single mutants; no misexpression is detectable in the discs from *phol* or *Ino80^KO^* single mutants or in the rudimentary eye antennal disc from *pho phol* double mutants. (C) Wing (left) and eye antennal imaginal disc (right) of third-instar larvae from *Sfmbt Ino80^KO^* double homozygous larvae. Note that the extent of Ubx (left) and Antp (right) misexpression is comparable to that seen in *Sfmbt* single mutants (compare with *Sfmbt* panels in A and B)

To further characterize the phenotype of *pho phol*, *Sfmbt* and *Ino80^KO^* mutants, we dissected wing imaginal discs and CNS tissues from wandering third-instar larvae and compared them to the same tissues from wild type, *pho* single or *phol* single mutant animals. In *pho phol* double mutant wandering larvae, all imaginal discs were consistently much smaller and misshaped compared to wildtype, or the *pho* or *phol* single mutants (Figure 2, A and B, Supplementary Figure S2A), whereas the size of the CNS was comparable to that in the wildtype or in *pho*, or *phol* single mutants (Supplementary Figure S2B). In *Sfmbt* mutants, the first and second leg imaginal discs were consistently reduced in size compared to wild-type, whereas the other discs and the CNS tissue showed no apparent size reduction but discs were morphologically distorted in each of the analyzed animals (Figure 2, A and B, Supplementary Figure S2, A and B). As expected from the wild-type morphology and size of *Ino80^KO^* mutant adults (Supplementary Figure S1B), the size of imaginal discs was comparable to that of wild-type larvae (Figure 2, A and B).

We next stained wild-type and mutant larvae with antibodies against the protein products of the PcG target genes *Ultrabithorax* (*Ubx*) and *Antennapedia* (*Antp*). *Ubx* was misexpressed in wing imaginal discs of *pho* and *Sfmbt* single and of *pho phol* double mutant larvae, as previously reported (Figure 2A; cf. Brown *et al.* 2003). *Antp* was misexpressed in the antenna primordium of the eye antennal imaginal disc of *pho* and *Sfmbt* single mutant larvae but we were unable to detect Antp in the poorly developed eye antennal disc of *pho phol* double mutant larvae (Figure 2B). As expected from the wild-type morphology of *Ino80^KO^* mutant adults, no misexpression of *Ubx* or *Antp* was detected in imaginal discs from *Ino80^KO^* homozygous larvae (Figure 2, A and B). To test for a possible genetic interaction between *Ino80* and *Sfmbt*, we generated *Sfmbt Ino80* double mutant animals. Larvae that were homozygous for both *Sfmbt^1^* and *Ino80^KO^* completed larval development and arrested during the early phase of puparium formation, like *Sfmbt* single mutants. *Ubx* misexpression in wing imaginal discs and *Antp* misexpression in eye-antennal discs from *Sfmbt Ino80^KO^* double mutants was comparable to that seen in *Sfmbt* single mutants (Figure 2C). Simultaneous removal of Ino80 and Sfmbt, therefore, did not enhance the Polycomb phenotype seen in *Sfmbt* single mutants.

**Figure 3:**
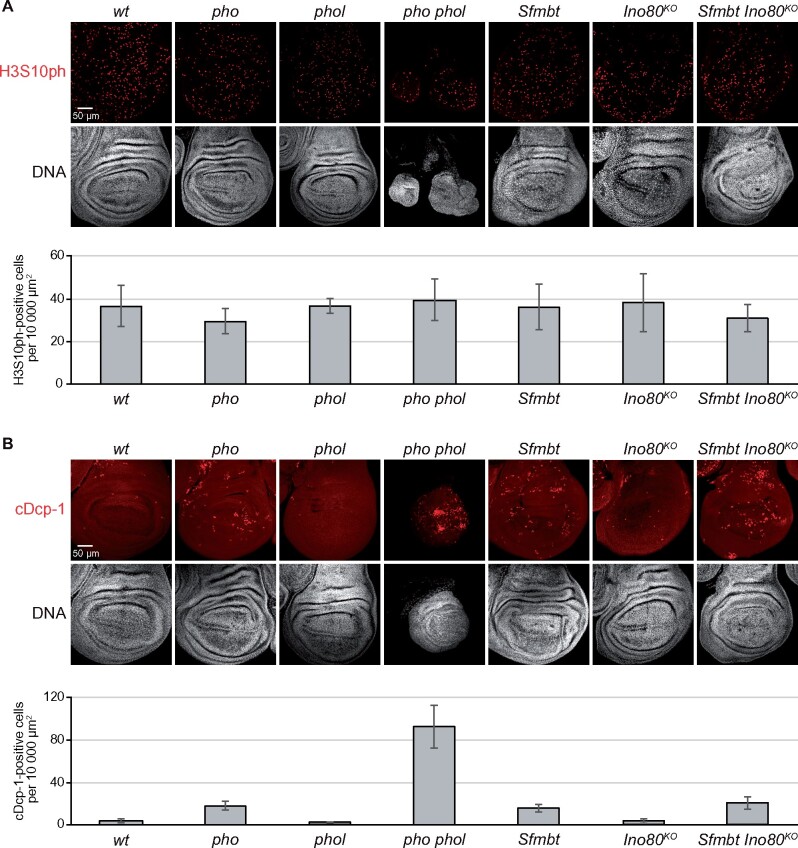
Cell proliferation is unimpaired in *pho phol*, *Sfmbt* or *Ino80* mutants but *pho phol* mutants show extensive apoptotic cell death. (A) Top: wing imaginal discs of third-instar larvae of the indicated genotypes, stained with antibody against H3S10ph and Hoechst (DNA) to label all nuclei. Below: Normalized number of H3S10ph-positive cells per area (10,000 µm^2^) of imaginal wing disc tissue in each genotype. For each genotype, bars represent the mean number and standard deviation of cells counted in discs from six different individuals. (B) Top: wing imaginal discs like in (A), stained with antibody against cDcp-1 and Hoechst (DNA). Below: Normalized numbers of the cDcp-1-positive nuclei per area (10,000 µm^2^) of imaginal wing disc tissue in each genotype. For each genotype, histogram bars represent the mean number and standard deviation of cells counted in discs from six different individuals.

We next investigated whether the small disc phenotype of *pho phol* mutants might be linked to a reduction in cell proliferation or to an increase in cell death. We first stained larval imaginal discs with antibodies recognizing histone H3 that is phosphorylated at serine 10 (H3S10ph), a modification that marks mitotic cells (Wei *et al.* 1998; Giet and Glover 2001). Quantitative analyses revealed that the fraction of H3S10ph-positive cells in *pho phol* mutants was not reduced compared to wildtype, and, moreover, was also undiminished in *pho*, *phol*, *Sfmbt* or *Ino80^KO^* single mutants (Figure 3A and Supplementary Figure S2A). Cells lacking both Pho and Phol protein or cells lacking Sfmbt protein, therefore, retain the capacity to proliferate.

We then stained the same tissues with antibodies recognizing the cleaved Death caspase-1 (cDcp-1). Cleaved Dcp-1, the active form of this effector caspase, is a universal marker of apoptotic cells (Song *et al.* 1997). In imaginal discs from wild-type animals, only a small number of cDcp-1-positive cells can be found in every disc (Figure 3B). In contrast, in *pho phol* mutant larvae, every wing disc shows a drastic increase in the number of cDcp-1-positive cells and these cells are often found in small clusters (Figure 3B). The occurrence of apoptotic cells in *pho phol* mutant larvae is particularly striking in the CNS and in the brain lobes, where in wild-type animals very little cell death is observed at this stage (Supplementary Figure S2B). We found that wing discs from *pho* single mutant and from *Sfmbt* mutant larvae also showed a larger fraction of apoptotic cells compared to wild-type but that the effect was much less drastic than in *pho phol* double mutants (Figure 3B). In *Ino80^KO^* mutant larvae, the fraction of cDcp-1-positive cells in discs was comparable to that in wild-type animals (Figure 3B). Finally, we found that the fraction of cDcp-1 positive cells in imaginal discs from *Sfmbt Ino80^KO^* double mutants was comparable to that in *Sfmbt* single mutants (Figure 3B). In conclusion, these experiments reveal that there is extensive cell death in *pho phol* mutant animals. Collectively, these data argue that cells lacking Pho and Phol are not impaired in their ability to proliferate but are severely compromised in their viability. A likely explanation for the small-disc phenotype in *pho phol* mutant larvae therefore is that the rate of cell death overrides the rate of cell proliferation and thereby precludes formation of normal-sized imaginal discs.

**Figure 4:**
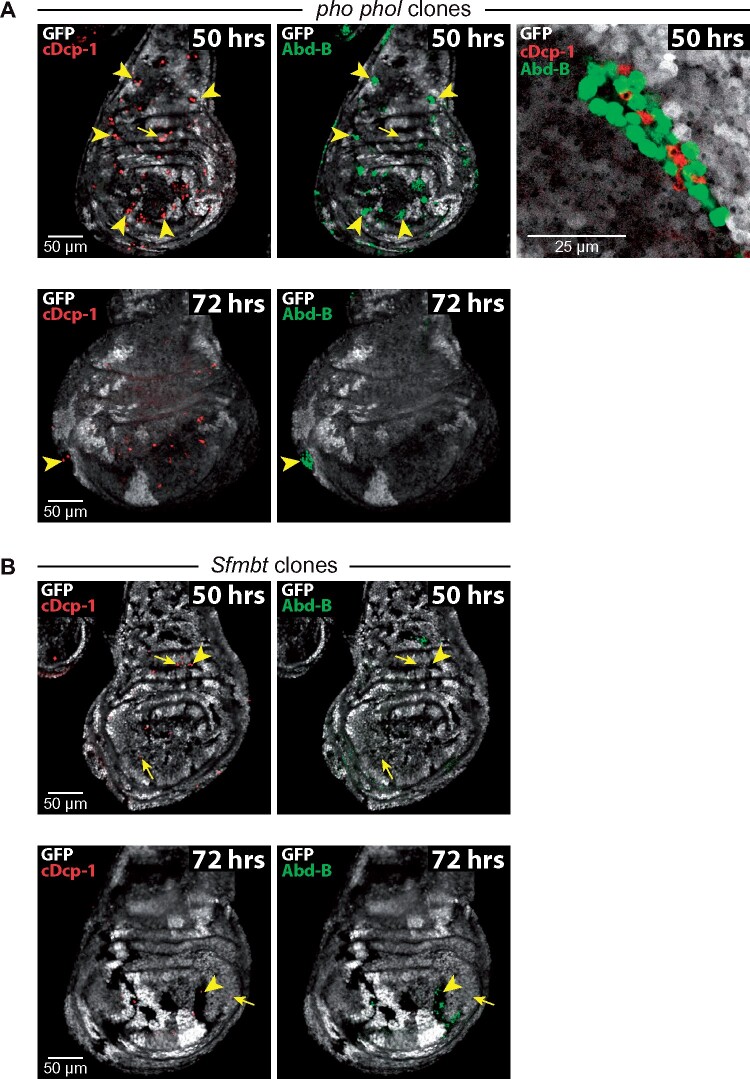
Pho and Phol but not Sfmbt are required for cell survival. (A) Top: wing imaginal discs of *phol^–/+^; pho^–/–^* animals with clones of *phol^–/–^; pho^–/–^* cells (in the text referred to as *pho phol* double mutant cells), analyzed 50 h after clone induction and stained with antibodies against cDcp-1 (red) and Abd-B (green), GFP is visualized in gray. The *phol^–/+^; pho^–/–^* cells carry one copy of the GFP marker gene (light gray), *phol^–/–^; pho^–/–^* clone cells are marked by the absence of GFP, whereas the *phol^+/+^; pho^–/–^* twin spot clone cells generated as the reciprocal recombination event during clone induction carry two copies of the GFP marker gene (bright gray). Left: a single imaginal wing disc is shown in two separate images to visualize cDcp-1 and Abd-B expression; note that many clones contain multiple cDcp-1-positive cells and also Abd-B-positive cells (arrowheads), the presence of cDcp-1 signal in GFP-positive cells (small arrow) is primarily because all cells in these animals are *pho^–/–^* (see Figure 3B). Note also that, unlike Ubx (Figure 2A), Abd-B is not misexpressed in *pho^–/–^* wing discs. Right: Image of a single *phol^–/–^; pho^–/–^* mutant clone in a wing imaginal disc 50 h after clone induction illustrates that clones are a mosaic of cells that are either Abd-B– or cDcp-1–positive. *Below*: wing imaginal disc from the same genotype as *above*, analyzed 72 h after clone induction, the two images visualizing cDcp-1 (left) and Abd-B (right) expression. Note that most *phol^–/–^; pho^–/–^* clone cells have been eliminated from the disc, the large *phol^+/+^; pho^–/–^* twin spot clones serve as reference. This disc contained a single surviving clone (arrowhead). (B) Top: wing imaginal disc of a *Sfmbt^–/+^* animal with clones of *Sfmbt^–/–^* cells, analyzed 50 h after clone induction and stained with antibodies against cDcp-1 (red) and Abd-B (green); clones are marked by the absence of GFP (visualized in gray). Note that *Sfmbt* mutant clones only show sporadic cDcp-1- positive cells (arrowheads), as also observed in wild-type tissue (small arrows, see also Figure 3B). Note that Abd-B is only misexpressed in a small fraction of clone cells, misexpression of Abd-B is therefore much less extensive than that of Ubx in *Sfmbt* mutant wing discs (Figure 2A) or in clones of *Sfmbt* mutant cells (cf. Frey et al., 2016). *Below*: wing imaginal disc of a *Sfmbt^–/+^* animal with clones of *Sfmbt^–/–^* cells as on top but analyzed 72 h after clone induction. Note that *Sfmbt* mutant clones continue to grow and that cDcp-1- positive cells in clone tissue (arrowhead) only occur sporadically, comparable to what is observed in wild-type tissue (small arrow).

To further investigate the requirement of Phol and Pho for cell viability, we generated clones of *pho phol* double mutant cells in larvae that were homozygous for *pho* and carried one wild-type allele of *phol* (*i.e.*, in *phol^–/+^; pho^–/–^* animals). We previously found that in this genetic background *pho phol* mutant cells initially proliferated to form clones but that 96 hours after clone induction such clones could no longer be detected (Klymenko *et al.* 2006). Here, we analyzed clones in wing imaginal discs 50, 72, and 96 hours after clone induction. The *pho phol* mutant cells were identified by the absence of a GFP marker gene, and we monitored cell death in the clones by staining the discs for cDcp-1. In addition, the discs were also stained with an antibody detecting Abd-B protein, the product of a classical PcG target gene that is normally not expressed in wing disc cells. Fifty hours after clone induction, most clones showed strong misexpression of Abd-B (Figure 4A, top row). In addition, a large fraction of these mutant clones also showed cDcp-1 signal (Figure 4A, top row). Superposition of the two signals revealed that the clones represented a mosaic of cells that either expressed Abd-B protein or were positive for cDcp-1 (Figure 4A, top row). This suggests a scenario where, after clone induction, the lack of Pho and Phol protein first results in a failure to maintain PcG repression and target genes like *Abd-B* become misexpressed but that this misexpression ceases as the cells then eventually enter apoptosis. In imaginal discs that were analyzed 72 hours after clone induction, we only found rare *pho phol* mutant clones in a fraction of the analyzed discs (Figure 4A, bottom row). As expected, no clones were detected if discs were analyzed 96 hours after induction (not shown; cf. Klymenko *et al.* 2006). How can the total elimination of *pho phol* mutant clone cells in this genetic background be explained given that in *pho phol* mutant animals, genetically identical *pho phol* mutant cells can still proliferate to form rudimentary imaginal discs? It is important to note that *phol^–/+^; pho^–/–^* animals reach the wandering third instar larva stage 120 hours AEL, with normal-sized wing imaginal discs (Figure 4A), whereas the *pho phol* animals shown in Figures 2, 3 and Supplementary Figure S2 reached the third instar larva stage only about 192 hours AEL. A likely scenario could therefore be that in imaginal discs from *phol^–/+^; pho^–/–^* animals, the *phol^–/–^; pho^–/–^* mutant clone cells—intrinsically already compromised for viability—are eliminated because of cell competition with their neighboring cells containing a *phol^+^* allele.

To complement these experiments, we also analyzed clones of *Sfmbt* mutant cells. We previously reported that *Sfmbt* mutant cell clones survive when induced in *Sfmbt^–/+^* animals and that such clones show misexpression of HOX proteins (Klymenko *et al.* 2006). Here, we stained imaginal discs with clones of *Sfmbt* mutant cells 50 hours and 72 hours after clone induction with antibodies against cDcp-1 and Abd-B. At both time points *Sfmbt* mutant clones showed misexpression of Abd-B in a few cells but no higher incidence of cDcp-1-positive cells compared to the neighboring wild-type tissue (Figure 4B). In clones, cells lacking Sfmbt, therefore, do not seem to be compromised in viability and growth.

## Discussion

Many protein complexes with dedicated biological activities contain subunits that function in multiple different protein assemblies. Consequently, animals lacking such shared subunits show phenotypes that are more complex because more than a single process has been disrupted. Among the proteins functioning in Polycomb repression in *Drosophila*, Pho and Phol fall into this category; they are subunits in both the PhoRC and the INO80 nucleosome remodeling complex (Klymenko *et al.* 2006). Unlike most other PcG proteins, Pho and Phol are essential for the survival of somatic cells. Here, we show that cells lacking Pho and Phol appear to proliferate normally but show a high incidence of apoptotic cell death. In contrast, *Sfmbt* mutant larvae show much less extensive cell death than *pho phol* mutants, and *Sfmbt* mutant cell clones in imaginal discs tissues survive. We show that cell division and survival is unaffected in *Ino80^KO^* mutant larvae and that *Drosophila* lacking zygotic expression of Ino80 are able to develop into morphologically normal, viable adults. Moreover, removal of both Ino80 and Sfmbt does not aggravate the cell death or PcG mutant phenotypes of *Sfmbt* mutants. This argues against the possibility that Sfmbt and Ino80 would cooperate with Pho and Phol through two redundantly acting pathways to preserve cell survival. It rather appears that removal of Pho and Phol function disrupts one or several processes needed for cell survival that do not require the function of PhoRC or the INO80 nucleosome remodeling complex.

How could Pho and Phol preserve cell survival? For the purpose of this discussion, we shall assume that Pho and Phol exert this function by regulating transcription of as yet unidentified subset of target genes. A few aspects of Pho and Phol should be noted here. First, absolute protein quantification studies measured that diploid nuclei in 2–4 hours old embryos contain about 20,000 molecules of Pho, about 20,000 molecules of Sfmbt, about 4000 molecules of Ino80 but only about 600 molecules of Phol (Bonnet *et al.* 2019). Nuclei from 14 to 16 hours old embryos contain about 2600 molecules of Pho and about 3000 molecules of Sfmbt, about 2000 molecules of Ino80, whereas Phol levels dropped below the limit of 100 molecules per nucleus that was needed for reliable detection and quantification (Bonnet *et al.* 2019). Considering that Pho and Phol have been found to associate either with Sfmbt or with the INO80 complex (Klymenko *et al*, 2006), these protein quantification data, therefore, argue against the idea of a large pool of free Pho and Phol protein. During both analyzed stages of embryogenesis, Pho and Sfmbt are present in near stoichiometric amounts and Phol is about 30-fold less abundant than Pho. Interestingly, Phol protein levels were not upregulated in cells in which Pho protein was depleted (Kahn *et al.* 2014). Considering that cell survival was only mildly compromised in *pho* single mutants, the low levels of Phol protein in *pho* mutants therefore must suffice to sustain cell viability. Second, since Pho and Phol act redundantly to ensure cell survival, one would expect the relevant target genes that need to be regulated to be co-bound by both Pho and Phol. Studies analyzing the binding profile of Pho found that the majority of Pho-bound sites correspond to Polycomb Response Elements (PREs) where Pho is co-bound with Sfmbt, PRC1 and PRC2, and the flanking chromatin is decorated with H3K27me3 (Klymenko *et al.* 2006; Papp and Müller 2006; Oktaba *et al.* 2008; Schuettengruber *et al.* 2009; Filion *et al.* 2010; Kharchenko *et al.* 2011; Kahn *et al.* 2014; De *et al.* 2016; Erceg *et al.* 2017; Bonnet *et al.* 2019). The binding profile of Phol has been analyzed in embryos (Schuettengruber *et al.* 2009) and in tissue culture cells (Kahn *et al.* 2014). Both studies reported that, like Pho, Phol is also bound at PREs of PcG target genes but that Phol in addition is also bound to other sites in the genome (Schuettengruber *et al.* 2009; Kahn *et al.* 2014). Together, these observations suggest that the genes which Pho and Phol need to regulate to preserve cell viability might be among the several hundred genes classified as PcG targets. In this context, it is important to emphasize that even though most genes cataloged as PcG targets show co-binding of PhoRC, PRC1, and PRC2 at PREs and H3K27me3 across their chromatin, genetic tests in different PcG mutants had found that at some of these target genes, only some components of PcG machinery are functionally needed for their repression (Beuchle *et al.* 2001; Oktaba *et al.* 2008; Gutiérrez *et al.* 2012). Transcriptome analyses to identify genes that are specifically deregulated in *pho phol* mutant larvae might be a strategy to identify the relevant target genes needed for cell survival, even though the interpretation of transcriptome changes will likely be challenging because of the complex tissue defects in these mutants. A third discussion point concerns the intriguing observation that the proapoptotic gene *reaper* (White *et al.* 1996) was identified as a PcG target in embryos and larvae (Oktaba *et al.* 2008; Zhang *et al.* 2008; Erceg *et al.* 2017). Previous studies showed that in early embryos ectopic expression of *reaper* can be induced by gamma-ray irradiation but that the gene becomes resistant to this induction in later-stage embryos (Zhang *et al.* 2008). This sensitive-to-resistant transition of *reaper* responsiveness to irradiation is accompanied by the binding of PcG protein complexes and trimethylation of H3K27 at the *reaper* locus (Zhang *et al.* 2008). Zhang and co-workers showed that in embryos with reduced PRC2 activity, the sensitive-to-resistant transition is delayed and also late-stage embryos showed at least some *reaper* expression upon gamma-ray irradiation (Zhang *et al.* 2008). Moreover, a *reaper-GFP* reporter gene has also been reported to be ectopically activated in clones of *polyhomeotic* mutant cells that form tumors in imaginal discs (Beira *et al.* 2018). The observation that the PcG machinery functionally represses the *reaper* gene raises the interesting possibility that lack of Pho and Phol might directly cause derepression of the *reaper* gene and thus account for the increase in apoptotic cell death in *pho phol* mutant animals.

Finally, we note that YY1 knock-out mouse embryos undergo implantation but then rapidly degenerated at that stage (Donohoe *et al.* 1999). Conditional removal of YY1 in B cells of developing mice (Trabucco *et al.* 2016; Kleiman *et al.* 2016) or in a vertebrate cell line (Sui *et al.* 2004) has been reported to induce apoptosis. Like Pho and Phol in flies, YY1 and its paralog YY2 may therefore act via a similar, conserved mechanism to preserve cell survival in mammals.

## Supplementary Material

iyab096_Supplementary_Data
