# Supplementary material for: Distinct requirements for Pho, Sfmbt, and Ino80 for cell survival in Drosophila
Source: Genetics. 2021 Jul 19;219(1):iyab096. doi: 10.1093/genetics/iyab096 (PMC8633127; doi:10.1093/genetics/iyab096)
Supplement: iyab096_Supplementary_Data [file iyab096_supplementary_data.pdf]

Figure S1

Drosophila with molecularly defined *Ino80*^{KO} deletion mutation develop into morphologically normal adults.

(A) Top: Representation of the *Ino80* genomic region in wildtype and in the *Ino80*^{KO} mutant. Coordinates of the base pairs on the edges of the *Ino80* transcription unit in the wildtype are indicated. Thin arrow shows direction of *Ino80* gene transcription. Dark grey boxes indicate protein-coding exons of *Ino80*, light blue arrows indicate the transcription units of the other genes located in the *Ino80* introns. Red box (not drawn to scale) indicates *DsRed* marker cassette integrated into the locus in the *Ino80*^{KO} mutant. Indicated genomic coordinates correspond to *Drosophila melanogaster* chromosome 3R (GenBank sequence AE014297.3). Below: Strategy for generating the *Ino80*^{KO} deletion allele by CRISPR/Cas9 genome editing. The 5' portion of the *Ino80* locus and of the neighboring *CG5361* gene are shown. Coordinates of the base pairs 5' to the cleavage sites generated by the Cas9-gRNA complexes are indicated. The coordinates of the homology arms borders cloned into the donor plasmid with the *DsRed* cassette are indicated. Two independently generated *Ino80*^{KO} alleles were validated by complete Sanger sequencing of the *DsRed* cassette insertion and its junctions with genomic regions, verifying that the promoter and coding region of *CG5361* were unaltered.

(B) Side view of wildtype (*wt*) and *Ino80*^{KO} homozygous male adults. Because of the size of the specimen, the images had to be assembled from four (*wt*) and five (*Ino80*^{KO}) separate brightfield micrographs that were taken in the same focal plane, using a 5x objective.

(C) Development of *Ino80*^{KO} homozygotes into adults is slightly delayed compared to wild-type animals. For each genotype, 600 24-27 h old first-instar larvae (input) were collected and reared in batches of 100 larvae in six separate vials at 25˚C. In each vial, the percentage of animals that eclosed as adults was determined over time. Points on the graph represent the mean and standard deviation of these percentages in individual vials.

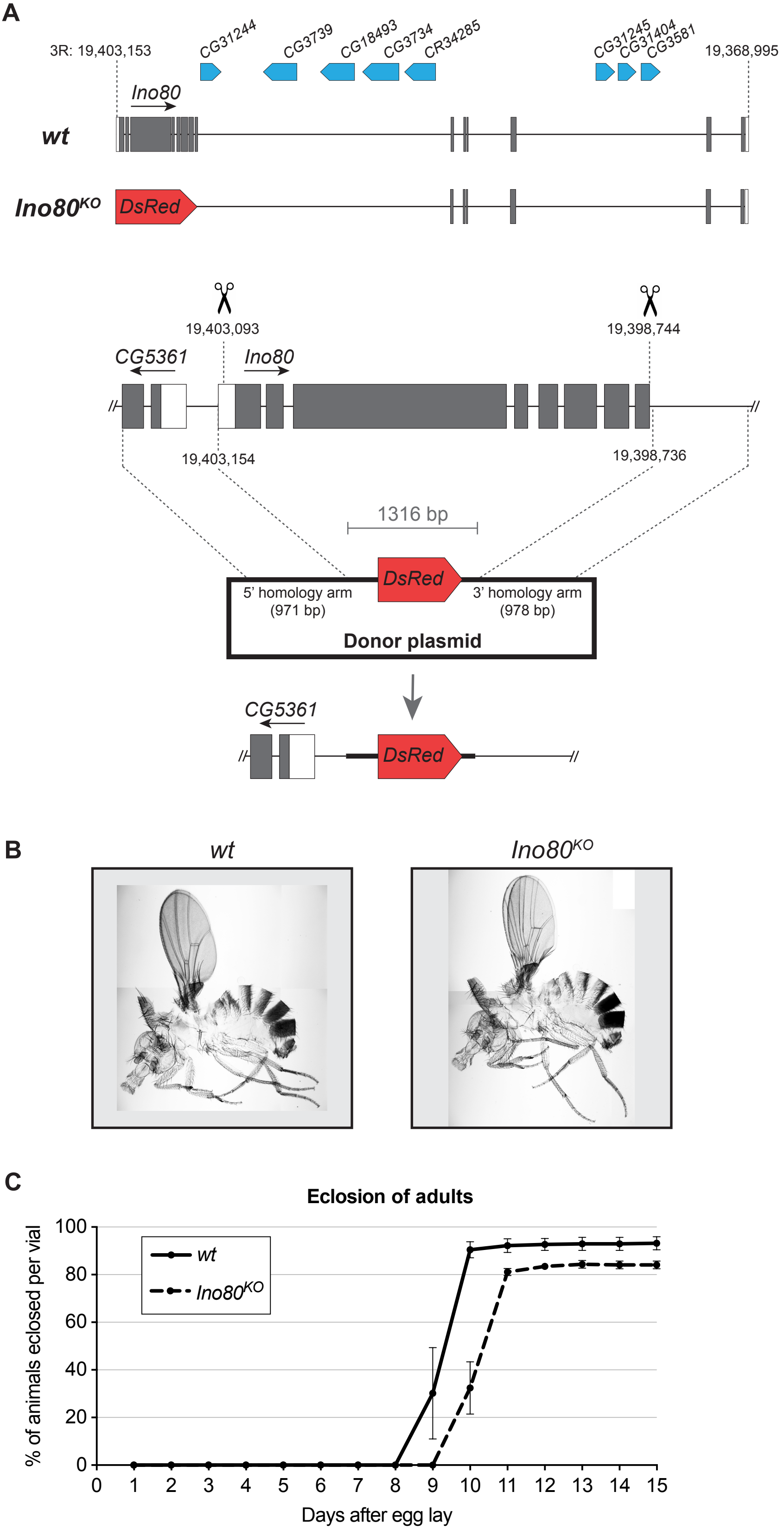
A
3R: 19,403,153
CG31244
CG3739
CG18493
CG3734
CR34285
CG31245
CG31404
CG3581
19,368,995
Ino80
wt
Ino80KO
DsRed
19,403,093
19,398,744
CG5361
Ino80
19,403,154
19,398,736
1316 bp
DsRed
5' homology arm
(971 bp)
3' homology arm
(978 bp)
Donor plasmid
CG5361
DsRed
B
wt
Ino80KO
C
Eclosion of adults
% of animals eclosed per vial
Days after egg lay
wt
Ino80KO
0
20
40
60
80
100
0 1 2 3 4 5 6 7 8 9 10 11 12 13 14 15

Figure S1

Figure S2

Cell proliferation is unimpaired in *pho phol* of *Sfmbt* mutants but *pho phol* mutants show extensive apoptotic cell death.

(A) Eye-antennal imaginal discs of third instar larvae from the same experiment like in **Figure 2B**, stained with antibody against H3S10ph and Hoechst (DNA). In eye discs of *wt*, *pho, phol*, and *Sfmbt* animals, the morphogenetic furrow with rows of mitotic cells is indicated with an arrowhead, the eye disc in *pho phol* mutants shows no obvious morphogenetic furrow. Note that in *pho phol* mutants, the density of H3S10ph-positive cells in other areas of the disc is comparable to that in the other genotypes; see **Figure 3A** for quantification.

(B) CNS tissues from third instar larvae from the same experiment like in **Figure 3B**, stained with antibody against cDcp-1 and Hoechst (DNA). Note that the CNS tissue in the *pho phol* mutant is not overtly smaller compared to the other genotypes but contains a large number of cDcp-1-positive cells. The size of pro- and/ or mesothoracic leg imaginal discs (L) is drastically reduced in *pho phol* mutant larvae compared to wildtype; note that in *Sfmbt* mutants these leg imaginal discs (L) are reduced in size and contain many more cDcp-1-positive cells than in wild-type animals.

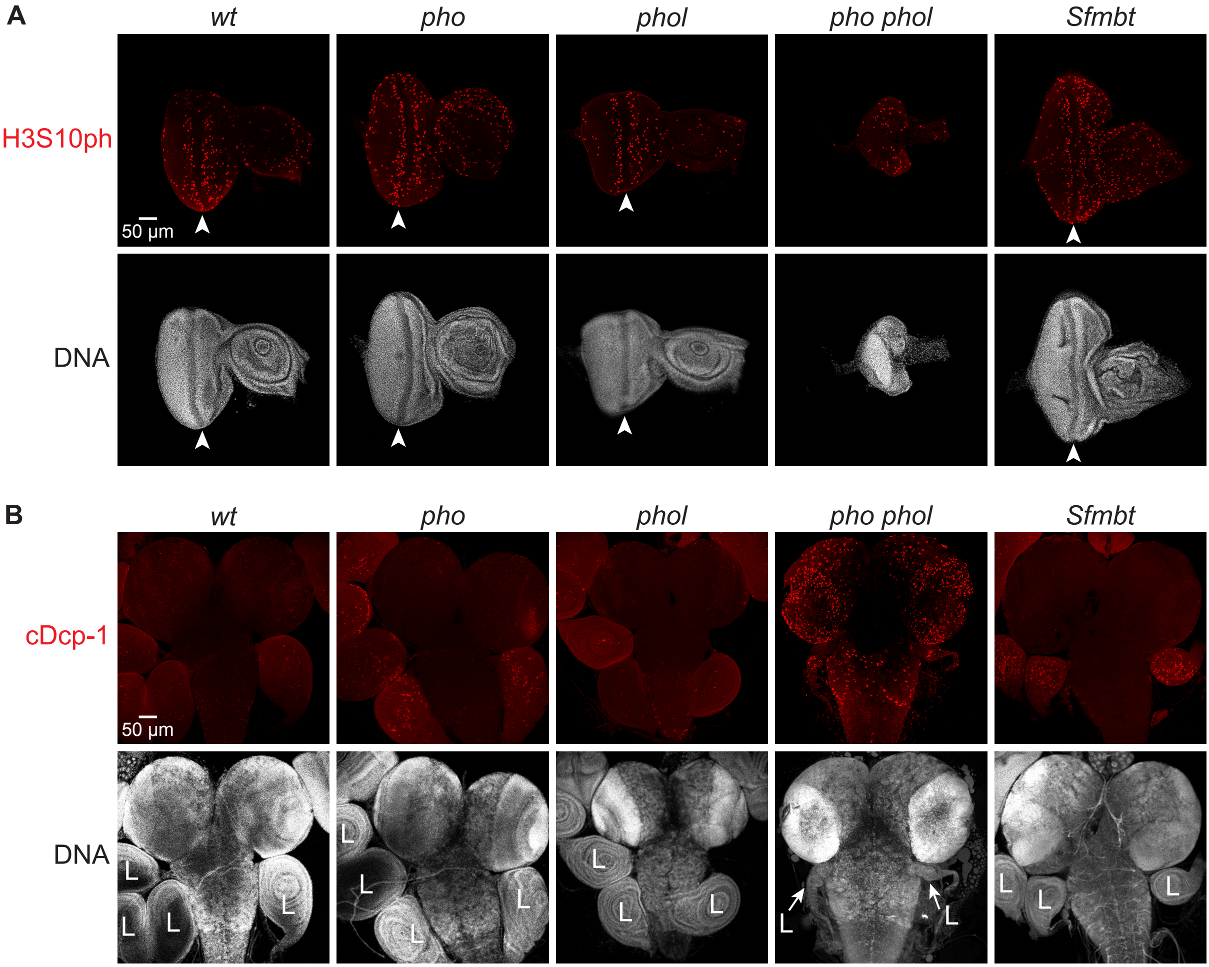
A
wt
pho
phol
pho phol
Sfmbt
H3S10ph
50 µm
DNA
B
wt
pho
phol
pho phol
Sfmbt
cDcp-1
50 µm
DNA
L

Figure S2
